# 

**Published:** 1911-05

**Authors:** 


### CONTENTS.

ORIGINAL COMMUNICATIONS
Conservative Surgery of the Uterine Appendages. Frank K. Boland, M.D., Atlanta, Ga. .... 55
Salvarsan, or "606," in the Treatment of Syphilis, with Report of Cases. By W. L. Champion, M.D., Atlanta, Ga. .... 59
Diseases of the Mouth. By Robin Adair, M.D., D.D.S. .... 67
The Traumatic Neurosis and Intelligent Psychotherapy. By Tom A. Williams, Washington, D. C. .... 72
Arteriosclerosis—Etiology, Diagnosis and Treatment. By J. H. Neall, M.D., Atlanta, Ga .... 79
Therapeutic Thoughts. By James Burke, M.D., Grimms, Wis. .... 87
A Suggestion in the Introduction of the Needle into the Vein in Making Intravenous Injections of "606." By Edgar G. Ballenger, M.D., and O. F. Elder, M.D., Atlanta, Ga. .... 89
EDITORIALS .... 94
NEWS AND NOTES .... 97
BOOK REVIEWS .... 108

